# Middle Meningeal Artery Embolization and the Treatment of a Chronic Subdural Hematoma

**DOI:** 10.7759/cureus.18868

**Published:** 2021-10-18

**Authors:** Likowsky L Désir, Randy D'Amico, Thomas Link, Danilo Silva, Jason A Ellis, Omer Doron, David J Langer, Rafael Ortiz, Yafell Serulle

**Affiliations:** 1 Department of Neurological Surgery, Lenox Hill Hospital, New York City, USA

**Keywords:** middle meningeal artery (mma), subdural hematoma (sdh), chronic subdural hematoma (csdh), middle meningeal artery embolization, prophylaxis

## Abstract

Chronic subdural hematoma (cSDH) is a common pathology that typically affects the elderly. It is believed to occur due to injury to the dural border cells, which creates an inflammation/proliferation reaction. Ineffective repair leads to the formation of a new external layer of cells and fragile capillaries, which damage easily and can worsen the condition. Conventionally, asymptomatic cSDH is managed by observation, and symptomatic cases are treated by surgical evacuation. Unfortunately, recurrence rates of the SDH following surgical evacuation can be high. One treatment option for SDH involves embolization of the middle meningeal artery (MMA). The MMA provides blood supply to the dura mater and feeds the membrane capillaries covering the SDH. MMA embolization blocks the blood flow to this system and can promote hematoma resolution. In this paper, we review the existing literature on MMA embolization and discuss the underlying pathophysiology of cSDH.

## Introduction and background

Chronic subdural hematoma (cSDH) is a common disease characterized by an organized collection of blood beneath the dural membrane. In the United States, from 2003 - 2016, the overall incidence of SDH more than doubled from 26.4 to 58.6 per 100,000, and the occurrence of cSDH with an ever-increasing elderly population is projected to be 60,000 new cases per year over the next 10 years [1-2]. Aside from age, other well-established risk factors include male gender, dependency on antiplatelet or anticoagulant medication, and chronic alcoholism [3]. With an increase in the aging population who are on numerous medications, along with the use of imaging technology, more cases of cSDH are being diagnosed each year. It is predicted that by 2030, there will be at least 60,000 new cases of cSDH every year [4].

The clinical diagnosis of cSDH can be challenging [5]. Its early stages are insidious and without clear symptoms. Frustratingly, later stages still comprise of nonspecific symptoms. These symptoms may include gait disturbance and falls (55.5%), mental deterioration (34.0%), limb weakness (34.0%), acute confusion (32.1%), headache (17.2%), drowsiness or coma (9.6%), speech impairment (5.7%), collapse (1.0%), and seizure (1.0%) [6]. Motor dysfunction may be present in the form of tremors or gait disturbances, and aphasia or paresthesia may also occur. Since the majority of patients are elderly, other age-related diagnoses may disguise the condition. These include dementia, Alzheimer’s disease, Parkinson’s disease, and normal-pressure hydrocephalus [7]. Consequently, many cases of cSDH are diagnosed in later stages. In later stages, patients may develop seizures or even hemiparesis [8].

cSDH has progressive long-term health effects. One study showed that the mortality rate due to cSDH was 13%, and at least 20% of patients had ‘poor discharge disposition’ due to the complications or comorbidities of cSDH that required long-term healthcare assistance [9]. Although the in-hospital mortality rate of cSDH is low, mortality rates at the end of six months to one year following presentation can be as high as 30%. This indicates that cSDH can act as a major event triggering the ‘end of life’ period in the older age group [10]. 

The complexity of cSDH merits review of the available literature and discussion of the underlying pathophysiology of cSDH in order to better understand the management of the disease.

## Review

Understanding the pathogenesis of chronic subdural hematoma

Traditionally, cSDH was mainly thought of as an accumulation of low-flow, bloody fluid collection of venous origin, built gradually and chronically in the subdural space as the result of a tear in the bridging veins en route to the dural venous sinuses [11].

However, several other factors appear to play a central role. Venous injury results in slow but immediate extravasation of blood into the subdural space, which would be visible on a head CT scan. This stands in contrast to clinical observations which show normal head CT scans obtained a few hours after trauma in patients who later develop cSDH [12]. In addition, the time frame in which the accumulation of venous blood corresponds to symptoms is variable and does not correlate directly with the time of the insult. For example, in the majority of cases, venous blood accumulation occurs within four to seven weeks following trauma [13]. Finally, on CT scan, cSDH are often visualized as a mass of mixed density with chronic and more acute components. This is inconsistent with venous bleeds that show up as an area of new hemorrhage [14].

Over the last few years, alternate pathophysiological mechanisms have been proposed. Chronic SDH is now considered a continuous process where inflammation and proliferation coexist. In a detailed review, Edlmann et al. described the various steps that may take place during the pathogenesis of cSDH [12]. It has been proposed that cSDH occurs not due to venous injury, but due to injury to a highly specialized group of cells called the dural border cells. The dural border lies on the inner side of the dura mater and is made up of modified connective tissue cells which, when healthy, are capable of depositing fibrocellular connective tissue. When these cells are injured, they elicit an inflammatory response in the region, which leads to fibrogenesis and angiogenesis [15]. In some instances, the inflammatory response is unable to repair the damaged layer of dural border cells. Instead, a new dural layer forms, itself comprising of two layers. The inner layer is continuous with the arachnoid and consists of collagen and fibroblasts and very few blood vessels. This layer does not contribute to the inflammatory process. The external layer is continuous with the dura and contains fibrocellular elements, as well as inflammatory cells, that worsen the inflammation. This layer also consists of many newly formed capillaries. These capillaries are highly permeable, with thin walls and numerous gap junctions, which makes them prone to injury [16].

Injury to the new, fragile capillaries leads to bleeding and an expansion of the cSDH. The bleeding is exacerbated by a hyperfibrinolytic state arising from raised levels of tissue plasminogen activator which is released from the external membrane and thrombomodulin which is released from injured vessel walls [17-18]. The injury thus triggers more inflammation and a vicious cycle is established. This allows for a chronic unabated growth of the SDH [19].

Conventional management of cSDH and its shortcomings

The nature of conventional management depends largely on whether the patient is symptomatic or asymptomatic, as well as hematoma size and compressive effect per imaging. In asymptomatic cases, observation and monitoring is advised. If the hematoma is small, spontaneous resolution may occur. Kim et al. studied 16 patients and showed that asymptomatic cSDH had a resolution rate of 81.3% [20]. However, even if asymptomatic, the size of the hematoma plays a role in treatment decisions. Hematomas that are less than 10 mm in diameter and have an associated midline shift of less than 5 mm are expected to resolve with conservative treatment [21]. Larger hematomas usually become symptomatic.

**Figure 1:**
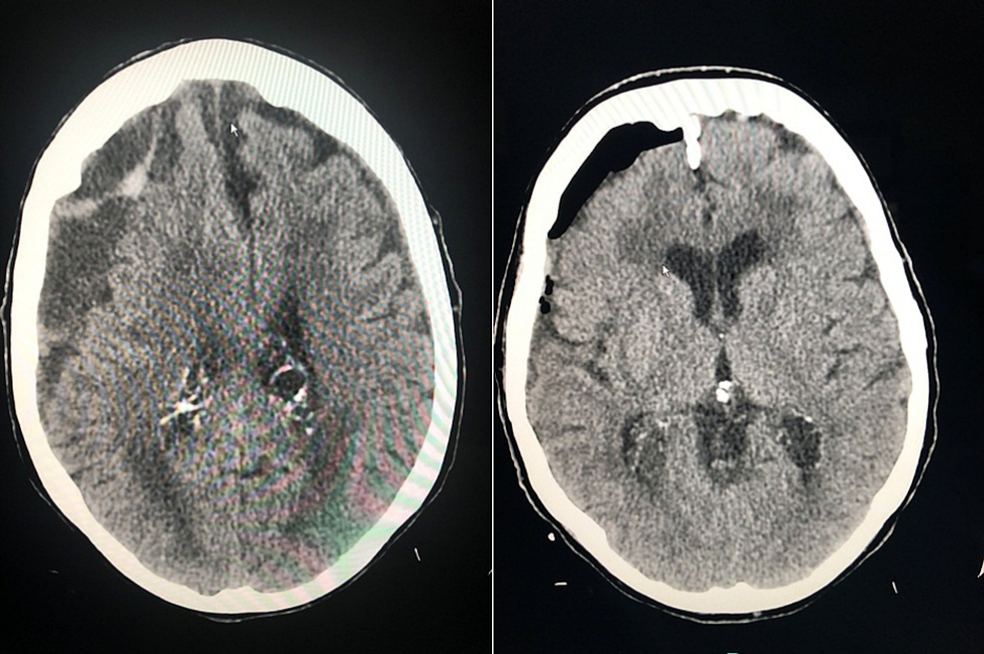
Pre- and post-surgical evacuation

In symptomatic patients, decompression of the hematoma is typically recommended which is typically achieved by surgical evacuation (Figure 1). Several different techniques can be used. The twist drill craniostomy can be performed at the bedside, and it consists of small burr holes (< 10 mm). When a bedside approach is not feasible, or for larger hematomas, a burr hole craniostomy is preferred. It consists of bigger burr holes (> 10 mm). Burr hole craniostomy is preferred by most surgeons. Craniotomy, which involves a larger bone window (> 30 mm), is warranted in cases with an acute component, or in recurrent cases when multiple membranes preclude drainage through a simple burr hole. Any form of hematoma evacuation is usually associated with the placement of a subdural drainage system for a brief postoperative period [22].

While surgical evacuation decompresses the hematoma and relieves symptoms, it does not eliminate the feeder capillaries or break the cycle of inflammation and bleeding. Therefore, the possibility for recurrence can be high.

In a meta-analysis of over 5,400 procedures by Ducret et al., the recurrence of cSDH was estimated to be 11.7% for burr hole craniostomies, 19.4% for open craniotomies, and 28.1% for twist drill craniostomies [23]. These procedures also carry risks of complications. The same meta-analysis showed that the complication rates for each of these procedures were 9.3%, 3.9%, and 2.5%, respectively. Complications include intracerebral hematomas, seizures, and tension pneumocephalus. Even in the absence of recurrence, there is a possibility of failure of cerebral re-expansion, which is largely due to the persistence of the dural membrane. Kung et al. established that the brain re-expanded only by about 41% on Day 14 and by 60% on Day 30. Re-expansion was reduced in larger bilateral hematomas [24]. Poor brain re-expansion in bilateral cSDH, compared with unilateral cSDH, may lead to brain parenchymal shift, damage to blood vessels, postoperative pneumocephalus, and cerebrospinal fluid (CSF) accumulation in the hematoma cavity, resulting in higher recurrence rates [25-27].

With a better understanding of the disease pathogenesis, attempts have been made to modify existing surgical procedures to address the outer membrane. In a meta-analysis of 17 clinical studies, Sahyouni et al. evaluated the effect of combined craniotomy and membranectomy in cSDH [28]. They found that the mean recurrence rate was 7.6%. The morbidity rate, defined as a major complication, disability, or poor health, was 6.9%, and the mortality rate was 3.7%. While the recurrence rate was slightly lower than conventional procedures, morbidity and mortality rates were comparable.

In an attempt to avoid the complications of surgery, certain non-surgical modalities have been proposed. The use of steroids has been suggested, with the assumption that its anti-inflammatory properties can help prevent the growth of the hematoma. Steroids have been shown to inhibit vascular endothelial growth factor (VEGF), which plays a role in the enlargement of the hematoma. Thotakura and Marabathina evaluated the effect of steroids in 26 patients with cSDH [29]. They found that 38.6% of patients did not show any improvement. An additional 19% showed initial improvement but developed recurrent symptoms after three weeks. The authors stated that steroids appeared to be more successful in patients with low-grade lesions.

Other treatment modalities have been tried but with little success. Atorvastatin can inhibit inflammation and promote vascular maturation. In one study, 25% of patients did not initially respond, while the 16.7% who responded required subsequent surgery [30]. Another retrospective study evaluated the use of etizolam, a platelet-activating factor receptor antagonist. This treatment reduced the need for surgery only in 46% of all patients [31]. One systematic review of all conservative methods of management concluded that they should be employed only in small, unorganized lesions where the neurological status was stable [32].

Middle meningeal artery (MMA) embolization in cSDH

The MMA, which provides blood supply to the dura mater, is believed to give rise to the capillary feeders that supply the cSDH.

Digital subtraction angiography (DSA) observations in cSDH patients reported a typical ‘‘cotton wool-like staining’’ of the distal vasculature on DSA, as well as contrast pooling, suggestive of leakiness and the immature nature of the neovessels [33}. Slow, continuous contrast injections created the appearance of contrast outlining the hematoma. Other findings included direct arterial feeders in what was traditionally thought to be a strictly ‘‘venous’’ pathology.

**Figure 2:**
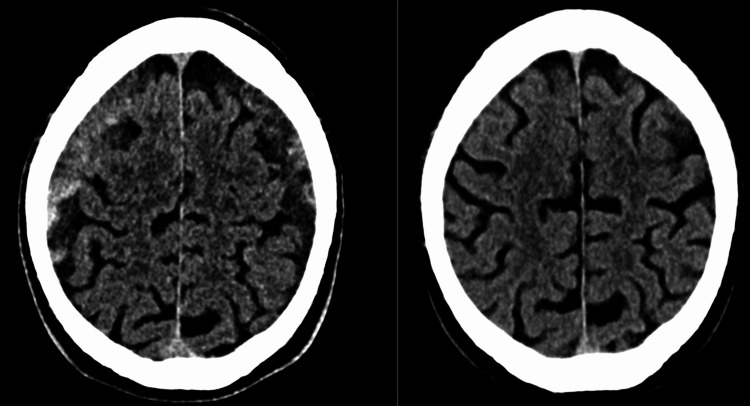
Pre- (left) and post-intervention (right) CT scan images of a patient who underwent craniotomy, as well as a middle meningeal artery (MMA) embolization. The right post-intervention image at three months indicates a clear resolution of the bilateral subdural hematoma (SDH).

Mino et al. evaluated four patients who presented with recurrent cSDH following single burr hole surgery [34]. They performed superselective angiography of the MMA artery in these patients. They found diffuse abnormal vascular stains around the MMA, which, according to them, represented the macrocapillaries on the external membrane of the cSDH. Following MMA embolization, they performed angiography of the external carotid artery. The abnormal vascular network around the MMA was no longer visible. Figure 2 displays pre- and post-intervention CT scan images of a patient who underwent craniotomy, as well as an MMA embolization.

Role of MMA embolization in prophylaxis against recurrent cSDH

Initially, MMA embolization was only employed when surgical treatment had failed. Mandai et al. were the first to report a case of MMA embolization for cSDH [35]. A 59-year-old man had developed refractory cSDH. The patient had a history of liver cirrhosis and had presented with decreased consciousness and progressive hemiparesis. Under local anesthesia, burr hole craniostomy was performed to evacuate the hematoma, and MMA embolization was performed to promote resolution and prevent a recurrence. The neurological deficit was reversed and no permanent deficit was noted.

Since then, several authors have used MMA embolization in combination with surgery for refractory cases that do not respond to surgical evacuation alone. In these reports, while surgical evacuation is used to relieve symptoms, MMA embolization is employed to prevent a recurrence. The rationale for utilizing this treatment was to decrease acute mass effect through direct surgical evacuation and endovascular MMA embolization to disrupt the reaccumulation chronic cycle. Okuma et al. demonstrated that MMA embolization alone could also be effective in cases of refractory cSDH [36]. Seventeen patients who underwent this procedure were followed for over two years with no signs of recurrence. There was also a significant improvement in neurological disability scores, as measured by the modified Rankin scale. Similarly, Shotar et al. found that postsurgical embolization of the MMA reduced the recurrence rate of cSDH from 14% to 4% [37].

Role of MMA embolization in the initial treatment of cSDH

Recent studies show that MMA embolization as initial single therapy has a role as primary therapy in select cases. Link et al. prospectively followed 50 patients who underwent MMA embolization for cSDH. Of these, 42 patients underwent embolization as first-line treatment, and eight patients were treated as recurrent cases after surgical evacuation had failed [38]. Four patients (8.9%) developed recurrence and surgical evacuation had to be performed, while 41 patients were stable and did not need further surgery. In 31 of these latter patients, imaging demonstrated a reduction of hematoma size that was greater than 50%. In the same study, MMA embolization was used as a prophylactic treatment after surgical drainage in 10 patients. In a recent systematic review, Court et al. assessed 18 articles in which a total of 190 patients had undergone MMA embolization. The authors estimated that resolution occurred in 96.8% of all cases, and there were no complications associated with the procedure [39]. A multicenter trial conducted by Kan et al. that involved 138 patients and 154 MMA embolizations employed endovascular therapy as first-line treatment in patients with either mild symptoms or midline shift of under 5 mm per CT. This therapy was used as a secondary treatment in patients not meeting the above criteria which comprised two-thirds of the patients. With regard to hematoma size, 70.8% of patients had a greater than 50% reduction while only nine patients (6.5%) required surgery. Hence, MMA embolization may offer a safe and effective alternative to conventional surgery for specific patients [40].

Comparison of MMA embolization with other treatment modalities

To date, three studies have conclusively proven that MMA is superior to other treatment modalities, both for initial and recurrent management [41-43].

Ban et al. compared conventional treatment modalities with MMA embolization in 541 patients [41]. Treatment failure occurred in only 1.4% of patients who underwent MMA embolization. In contrast, the failure rate was 27.5% among patients who underwent surgical removal.

Kim compared MMA embolization against burr hole craniostomy for patients who had already undergone prior surgical evacuation of cSDH [42]. They found that MMA embolization was successful in all except one case (3.8%), which eventually underwent spontaneous regression. On the other hand, the recurrence rate in patients who underwent craniostomy was 33.3%. Repeat craniostomy was performed in 20.8% of patients, while craniotomy was needed in 12.5% of the patients. The author recommended that MMA embolization must be considered as the preferred treatment in recurrent cSDH.

In another comparative study, Matsumoto et al. evaluated different methods of management in patients with refractory cSDH, which is defined as those presenting with two or more recurrences. Patients who underwent burr hole craniostomy alone had a recurrence rate of 25% [43]. No recurrence was seen in patients who were treated with MMA embolization or craniotomy with outer membranectomy. The authors recommended that MMA embolization should be considered for refractory patients with small hematomas. However, for large, organized hematomas, craniotomy with outer membranectomy may be a more suitable option.

Based on the above studies and six other single-arm studies, Srivatsan et al. performed a meta-analysis to assess the efficacy of MMA embolization [44]. They found that the odds of developing a recurrence after MMA embolization were 91.3% less than conventional methods. The overall recurrence rate was estimated to be 3.6%.

Technical aspects of MMA embolization

MMA embolization is a minimally invasive procedure that can be performed either under moderate sedation or general endotracheal anesthesia, depending on the preference of the surgeon. Access to the arterial system is usually obtained through the common femoral artery, although the radial route has been used in these cases more recently [45]. The diagnostic catheter is advanced to the common carotid artery (CCA), where a CCA injection is done first. Importantly, the vascular supply of the convexital cSDH that is being treated should correspond to the MMA distribution that is planned for embolization. Under roadmap guidance, a microcatheter is then advanced and navigated selectively into the MMA [46].

**Figure 3:**
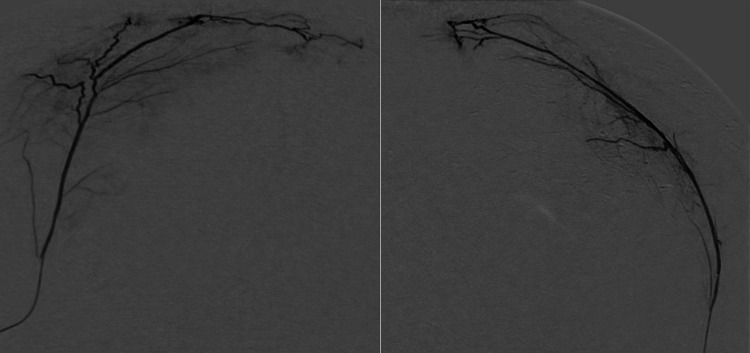
Frontal (left) and lateral (right) selective middle meningeal artery (MMA) angiograms demonstrating areas of contrast blush in the subdural hematoma

MMA angiography is then performed to visualize the MMA anatomy and to determine which of its branches are feeding the external membrane of the cSDH. Figure 3 shows frontal and lateral selective MMA angiograms demonstrating areas of contrast blush in the subdural hematoma.

Intimate knowledge of the external carotid anatomy, which is not demonstrated often in everyday procedures in neurointerventional surgery, is imperative. Visualization of the branches of the MMA and its anastomotic connections is crucial before an embolization is performed, as the MMA is feeding several cranial nerve nuclei and connects several external carotid artery/internal carotid artery (ECA-ICA) anastomoses which function as “trap doors” that the surgeon must recognize and avoid.

The MMA originates in the infratemporal fossa from the first part of the internal maxillary artery [46]. It enters the skull base at the middle meningeal fossa through the foramen spinosum. It divides into four branches from anterior to posterior - the sphenoidal, frontal, parietal, and petrosquamosal branches.

The MMA anastomoses with the anterior falcine branch of the ophthalmic artery and with the meningeal branches of the cavernous branch of the ICA [48]. Mutually anastomotic branches, such as the variant cavernous branch and the middle meningeal branch within the inferolateral trunk (ILT), possibly supply the Meckel's cave and its associated nerves. Since the MMA can connect with the ILT, it is also a possible “dangerous anastomosis” with the ICA. An extremely rare connection between the ILT and ICA is possible through a very long route of the sphenoid branch connection to the recurrent meningeal artery. Operators should also avoid the petrosal branch of the MMA and, if a prominent branch is identified, must navigate the microcatheter past this take-off and avoid reflux into it, as this branch can supply the vasa nervorum of cranial nerve VII within the petrous bone, as well as the greater petrosal nerve.

In addition, the microcatheter must be advanced to the sphenoid ridge to avoid influx into the meningo-ophthalmic branches of the MMA and prior to the anastomotic junctions [49]. If embolization is performed too close to the anastomoses, there is a risk of blindness or stroke. Therefore, if the vessel is too diminutive to navigate distally, the procedure should be reconsidered.

Most of the studies on MMA embolization have used polyvinyl alcohol (PVA) particles suspended in an opaque carrier solution [35, 38, 41]. Some authors have also used coils for embolization [50]. Hashimoto et al. suggested that this approach may be feasible when there is a likelihood of anastomosis with the ophthalmic artery in the proximal part [33]. However, Fiorella and Arthur suggested that liquid embolic agents could be considered as an alternative, as they are faster and simpler to use [21]. They pointed out that PVA particles could only be viewed on the angiogram due to the opaque carrier, which made distal penetration and reflux difficult to observe. However, since liquid agents are inherently radio-opaque, visualization would be easier and reflux better managed. Liquid agents, like cyanoacrylates and sodium tetradecyl sulfates, have been shown to produce better and more durable results. However, they have the potential to cause inflammatory reactions and even tissue death. These factors must be thoroughly evaluated before such agents are considered for MMA embolization. Recently, Rajah et al. have successfully used both non-adhesive (Onyx®) and adhesive (cyanoacrylate) liquids for MMA embolization in the management of cSDH [45].

## Conclusions

MMA embolization is a promising technique for the treatment of cSDH and can be used in both primary and recurrent cases. Although a few comparative studies have proven its superiority, large-scale randomized controlled trials with longer follow-up periods are essential to provide stronger evidence. Also, future research should focus on evaluating different types of agents that may be used for the embolization itself. Traditionally, most cSDH patients present in the ED for neurosurgical evaluation or are seen by primary care physicians, and the option of endovascular therapy must be brought to their awareness. The management of cSDH patients is complex and includes observation, medical therapy, and endovascular and surgical options. Therefore, physicians must be cognizant that successful implementation of this modality requires a team approach, as the role of MMA embolization in the treatment of cSDH will continue to evolve.
